# Functional Characterization of Cultured Keratinocytes after Acute Cutaneous Burn Injury

**DOI:** 10.1371/journal.pone.0029942

**Published:** 2012-02-16

**Authors:** Gerd G. Gauglitz, Siegfried Zedler, Felix v. Spiegel, Jasmin Fuhr, Guido Henkel v. Donnersmarck, Eugen Faist

**Affiliations:** 1 Department of Dermatology and Allergy, Ludwig-Maximilian University, Munich, Germany; 2 Department of Surgery, Ludwig-Maximilian University, Munich, Germany; 3 Burn Unit, Department of Plastic and Reconstructive Surgery, Community Hospital Bogenhausen, Munich, Germany; Ludwig-Maximilian-University, Germany

## Abstract

**Background:**

In addition to forming the epithelial barrier against the outside environment keratinocytes are immunologically active cells. In the treatment of severely burned skin, cryoconserved keratinocyte allografts gain in importance. It has been proposed that these allografts accelerate wound healing also due to the expression of a favourable - keratinocyte-derived - cytokine and growth factor milieu.

**Methods:**

In this study the morphology and cytokine expression profile of keratinocytes from skin after acute burn injury was compared to non-burned skin. Skin samples were obtained from patients after severe burn injury and healthy controls. Cells were cultured and secretion of selected inflammatory mediators was quantified using Bioplex Immunoassays. Immunohistochemistry was performed to analyse further functional and morphologic parameters.

**Results:**

Histology revealed increased terminal differentiation of keratinocytes (CK10, CK11) in allografts from non-burned skin compared to a higher portion of proliferative cells (CK5, vimentin) in acute burn injury. Increased levels of IL-1α, IL-2, IL-4, IL-10, IFN-γ and TNFα could be detected in culture media of burn injury skin cultures. Both culture groups contained large amounts of IL-1RA. IL-6 and GM-CSF were increased during the first 15 days of culture of burned skin compared to control skin. Levels of VEGF, FGF-basic, TGF-ß und G-CSF were high in both but not significantly different. Cryoconservation led to a diminished mediator synthesis except for higher levels of intracellular IL-1α and IL-1ß.

**Conclusion:**

Skin allografts from non-burned skin show a different secretion pattern of keratinocyte-derived cytokines and inflammatory mediators compared to keratinocytes after burn injury. As these secreted molecules exert auto- and paracrine effects and subsequently contribute to healing and barrier restoration after acute burn injury therapies affecting this specific cytokine/growth factor micromilieu could be beneficial in burned patients.

## Introduction

Loss of the integrity of large portions of the skin as a result of burn injury may lead to major disability or even death. As the skin forms an active barrier protecting our organism from the outside environment rapid restoration of the epidermal barrier is of vital relevance after acute burn injury. Thus, appropriate wound care is mandatory and various treatment modalities have been utilized to improve and accelerate wound healing. In the past decades, the increasing knowledge of the molecular and cellular mechanisms underlying wound repair and regeneration has led to extensive usage of growth factors in wound care [1]–[8]. Growth factors and cytokines play major roles in the well-orchestrated integration of the complex biological and molecular events underlying cutaneous wound healing, including cell migration and proliferation, extracellular matrix deposition, angiogenesis and tissue remodeling [9]. However, the clinical effects of the topical application of single growth factors to accelerate wound healing have been discouraging: On the one hand due to the complexity of the wound healing cascade and on the other due physical inhibition and biological degradation of topically applied factors [10]. The development of gene transfer technology promised to overcome the limitations associated with the (topical) application of recombinant proteins by delivering the respective growth factor directly to the wound bed [11]. Also, stem cells - due to their pluripotency and their growth potential - make them a potentially useful vehicle for gene delivery to injury site [10]. Still, the use of stem cell technology is far from a therapeutic application to date.

The epidermal skin barrier is formed by keratinocytes which secrete a multitude of biological active molecules contributing transiently to inflammatory responses and wound healing. In particular, keratinocytes have been shown to control the behaviour of fibroblasts during wound healing through the secretion, activation or inhibition of cytokines and growth factors such as TGF-β [12]. In the treatment of severely burned patients autologous keratinocyte-sheets but also (cryoconserved) allografts are used. It is supposed that in comparison to keratinocytes sheets these allografts accelerate wound healing possibly due to the expression of favourable cytokines und growth factors. However, the mechanisms of burn wound healing after allograft transplantation are not well characterized.

## Materials and Methods

### Patients

Seventeen severely burned patients, admitted to the Department of Burns, Community Hospital Bogenhausen, Munich, Germany, between 2002 and 2005, were enrolled in this prospective study. Inclusion criteria included: Admission within 24 hours post burn injury, burns covering more than 25% of the total body surface area (TBSA), <70 years of age, written consent to the experimental protocol. The study design was approved by the local ethics committee at the Ludwig-Maximilian University, Munich, Germany. 17 randomly selected female patients who underwent breast reduction surgery served as controls. On admission, the extent and degree of burn was assessed and recorded on a standard Lund and Browder chart. Information recorded at the time of admission included burn related (date and mechanism) as well as demographic data (age and gender). All patients were treated in our burn intensive care unit according to standardized protocols. Sepsis was defined as a positive blood culture or pathologic tissue culture identifying the pathogen during hospitalization or at autopsy, in combination with at least 3 of the following: leucocytosis or leucopenia (>12,000 or <4,000), hyperthermia or hypothermia (>38.5 or <36.5°C), tachycardia (>150 BPM in children), refractory hypotension (systolic BP<90 mmHg), thrombocytopenia (platelets <50,000/mm3), hyperglycemia (serum glucose >240 mg/dl), and enteral feeding intolerance (residuals >200 cc/hr or diarrhea >1 L/day).

### Isolation and culture of human keratinocytes

**Figure 1:**
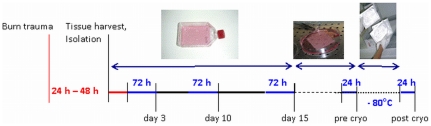
Time line and study design.

Tissue specimens were obtained during surgical debridement close (approximately 5 cm) to full-thickness burn wounds. Tissue from randomly selected female patients who underwent breast reduction surgery served as a control. Epidermal keratinocytes were isolated starting immediately after tissue harvest following the method of Rheinwald and Green [13]. In brief, harvested tissue was trypsinized (Trypsin 0,05%/EDT 0,02%, Gibco BRL, Invitrogen GmbH, Karlsruhe, Germany) for 12 to 18 hours and dermis was mechanically removed. Obtained cells were resuspended in Dulbecco' s modified Eagle' s medium (DMEM)/HAMS-F-12 media (Biochrom AG Seromed, Berlin, Germany), centrifuged at 200 g, resuspended in 20 ml of DMEM/HAMS-F-12 media supplemented with L-Glutamine, NaHCO_3_, EGF, FCS, gentamycin, insulin and hydrocortisone and seeded in cell culture flasks at 2×10^6^ each flask. Culture medium was collected and frozen in liquid nitrogen for further analysis after 72 hour incubation periods prior to day 3, 10 and 15 (Figure 1). At day 15 confluent cells were washed with dispase solution (2,5 mg/ml DMEM, Roche Diagnostics GmbH, Mannheim, Germany) for 20 minutes at 37 C and 5% CO_2_. A piece of Grassolind (Paul Hartmann AG, Heidenheim, Germany) measuring 75 cm^2^ was applied to the cell layer and attached by hemoclips. 24 hours later the culture media was collected (pre cryo) before cell sheets were frozen in MEM Earle supplemented with 20% FCS and 10% DMSO at −80°C. Later on sheets were thawed and re-cultured in DMEM. 24 hours thereafter culture media was collected and frozen for further processing (post cryo, Figure 1).

### Cellular morphology and immunhistochemistry

For routine light microscopy, cytospins from cultured keratinocytes from normal and burned skin were fixed in 10% formalin and stained with hematoxylin and eosin (at day 10). Immunohistochemistry was used to detect cytokeratin, vimentin and other proteins in order to allow comparison of cellular composition in allografts prepared from discarded tissue following breast reduction versus autologous cultured keratinocytes. For this, cytospins were incubated with the respective primary monoclonal antibodies (listed in Table 1). After frequent washings, a biotinylated rabbit anti-rat secondary antibody or a rabbit anti-mouse secondary antibody was applied respectively. After rinsing, enzyme conjugate was applied for 10 min. Final incubation included Substrate–Chromogen Mixture (3-amino- 9-ethylcarbazole, AEC) and positive staining revealed a red precipitate.

**Table 1:** Antibodies used for immunohistochemical analyses.

Antigen	Anti-	Isotype	Product #	Company	conc.
Control	MOPC21	IgG1	M9269	Sigma	1 mg/ml
Control	UPC-10	IgG2a	M9144	Sigma	1 mg/ml
Cytokeratin 2,6,8,10,11,18,19	Kl-1	IgG1	1918	Immunotech, Marseille	126,5 ng/ml
Vimentin	Vim 3B4	IgG2a	M7020	Sigma	60 µg/ml
CD 45	T 200	IgG1	M0701	Sigma	0,45 mg/ml
PECAM-1/CD 31	1F11	IgG1	2052 Klon 1F11	Immunotech, Marseille	200 ng/ml
DC-Sign/ CD 209	DCN46	IgG2b	551249	BD Biosciences	31,25 µg/ml

### Cytokine and growth factor measurements

The Bio-Plex Human Cytokine 17-Plex panel was used with the Bio-Plex Suspension Array System (Bio-Rad, Hercules, CA) to profile expression of interleukin (IL)-1α, IL-1 receptor antagonist (IL-1Ra), IL-1β, IL-2, IL-4, IL-6, IL-10, interferon-gamma (IFN-γ), tumor necrosis factor alpha (TNF-α), Vascular endothelial growth factor (VEGF), fibroblast growth factor basic (FGF-basic) granulocyte colony-stimulating factor (GCSF) and granulocyte-macrophage colony-stimulating factor (GM-CSF). The assay was performed according to the manufacturer's instructions. Briefly, cell culture medium was centrifuged at 4500 rpm for 3 minutes at 4°C. Samples were then incubated with microbeads labeled with specific antibodies to one of the aforementioned cytokines for 30 minutes. Following a washing step, the beads were incubated with the detection antibody cocktail, each antibody specific to a single cytokine. After another wash step, beads were incubated with streptavidin-phycoerythrin for 10 minutes, again washed and the concentration of each cytokine was determined using the array reader. Transforming growth factor beta-1 (TGF-β1) was determined by double-sandwich, enzyme-linked, immunosorbent assays (Biosource Europe SA, B-1400 Nivelles, Belgium) according to the protocol of the manufacturer in a microplate reader (Vmax, Molecular Devices Corporation, Sunnyvale, CA, USA) at 450 nm.

### Statistical analysis

Paired and unpaired Student's t-test and Mann-Whitney tests were used where appropriate. Data are expressed as means ±SD or SEM as indicated. Differences were considered significant at a p value of <0.05.

## Results

### Patient characteristics

Characteristics of burn patients and controls at the time of hospitalization are listed in Table 1. Patients were on average 41 years old and suffered from severe burn injury involving 42% of TBSA and third-degree burn involving 28% of TBSA. There were more male patients than female patients in the experimental group. During acute hospitalization, 24% of the patients suffered from inhalation injury, sepsis occurred in 29% and multi-organ failure (MOV) in 18% of patients. 24% of our patients subsequently died from their injuries (Table 2). 17 randomly selected female patients aged 20 to 50 years (mean 37±7 years) who underwent breast reduction surgery served as controls.

**Table 2:** Patient characteristics (n = 17).

*Patient characteristics*	*Mean ± SEM*
Age (years)	41±4
Gender (F/M)	3/14
TBSA (%)	42±2
3^rd^ degree (%)	28±4
MOV (%)	18
Inhalation injury (%)	24
Sepsis (%)	29
Non-survivors (%)	24

Randomly selected female patients who underwent breast reduction surgery served as a control population (n = 17; Age 37±7 years).

### H&E stainings and immunhistochemistry

**Figure 2:**
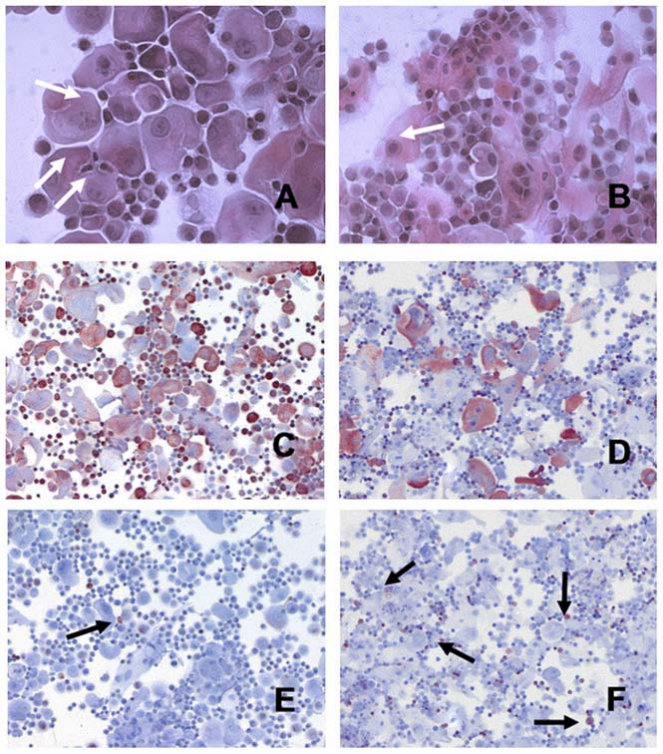
Morphological characterization of keratinocytes from acute burn injury. H&E stainings revealed a significantly increased proportion of terminally differentiated cells (white arrows) in allogenic cultured epithelial grafts (Fig. 2A) from breast reduction material in comparison to cells from autologous cultured epithelial grafts from burn patients (Fig. 2B). In stainings with the monoclonal antibody KL-1 significantly more terminal differentiated keratinocytes in allogenic grafts (Fig. 2C) were detected compared with autologous cultured epithelial grafts (Fig. 2D). More vimentin-positive cells are present in autologous cultured epithelial grafts (Fig. 2F) when compared to allogenic sheets (black arrows, Fig. 2E).

H&E stainings revealed a significantly increased proportion of terminally differentiated cells (white arrows) in allogenic cultured epithelial grafts from control tissue (Fig. 2A) in comparison to cells from autologous cultured epithelial grafts from burn patients (Fig. 2B). In stainings with the monoclonal antibody KL-1 (which marks the cytokeratins 2, 6, 8, 10, 11, 18 and 19 and especially K 6, 10 and 11 are expressed by terminally differentiated keratinocytes) significantly more terminal differentiated keratinocytes in allogenic graft (Fig. 2C) were detected compared with autologous cultured epithelial grafts (Fig. 2D). Vimentin is expressed by mesenchymal cells (e.g. fibroblasts) but also by basaloid keratinocytes. More vimentin-positive cells were present in autologous cultured epithelial grafts (Fig. 2F) when compared to allogenic sheets (Fig. 2E). No CD-209, 1F11 or T-200 (CD45) or MOPC-21 expressing cells could be detected in either group indicating the absence of dermal dendritic cells, macrophages, monocytes, endothelial cells and leukocytes (data not shown).

### Cytokine expression profiles

**Figure 3:**
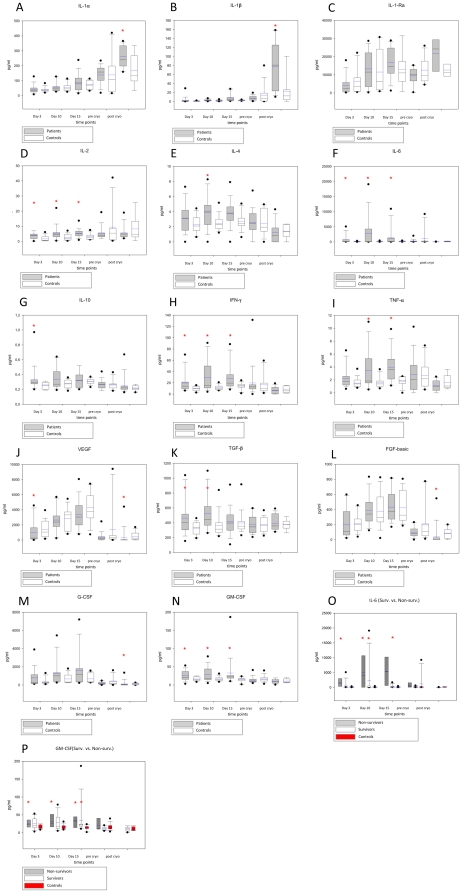
Cytokine profiles in keratinocytes cultures from burn injuries. Fourteen cytokines secreted by keratinocytes and measured in this study were significantly altered in keratinocytes cultures from sites of burn injury. Particularly levels of IL-2, -4, IL-6, -10, IFN-γ, TGF-β1 and TNFα were significantly altered in response to burn injury as compared to controls. Histograms depict culture media concentrations of the respective cytokine at the time of analysis. Bars represent means; error bars correspond to SEM. Asterisks denote statistical difference between cultures from burned skin vs. control skin. (* p<0.05).

No significant differences between groups could be measured in culture supernatants for IL-1α within the first 15 days of culture (Fig. 3A). During cell culture, levels of IL-1β were barely detectable at all time points measured (Fig. 3B). In contrast, IL-1RA was detected at high concentrations in culture media of both patients and controls peaking at day 15 (16690,69±2258,85 pg/ml vs. 12807,68±3016,70, respectively) without revealing any significant difference between patients and controls (Fig. 3C). Analyses of keratinocyte culture media revealed significantly elevated levels of IL-2, -4, -6, -10, IFN-γ, TNF-α and GM-CSF (Fig. 3D–I, N) at most of the first three time points studied in the patient group compared to control patients. However, expression of most of the respective cytokines was generally very low. Expression levels of these cytokines did not significantly differ when comparing non-surviving patients with survivors of the burn trauma (data not shown).

IL-6 and GM-CSF were significantly increased during the first 15 days of culture after acute burn injury compared to control skin (Fig. 3F, N) as a result of significant higher expression in non-survivors, Fig. 3O, P). Concentrations of IL-6 increased early after burn injury peaking at 10 days post injury (2874,04±1352,89 pg/ml) while culture media analysis of the control group demonstrated basic levels during the 15 day culture period (with 69,35±21,82 pg/ml; 60,37±28,19 pg/ml, and 98,28±35,38 pg/ml, respectively). Noteworthy, when differentiating between survivors and non-survivors, keratinocytes of patients that did not survive the burn trauma revealed significantly increased secretion of IL-6 at the first three time points measured when compared to the survivor group. The expression profile of GM-CSF revealed a similar pattern however, differences between groups were, although statistically significant, not as pronounced as with IL-6.

Levels of VEGF, TGF-ß, FGF-basic und G-CSF could be detected at relatively high concentrations, however only moderate differences could be detected comparing the two collectives (Fig. 3J–M). Interestingly, at 3 and 10 days post burn, keratinocytes isolated close to a burn wound did secrete significantly elevated levels of TGF-β1 compared to normal controls (e.g. 526,43±61,16 pg/ml and 362,59±38,50 pg/ml at day 10, respectively). No differences could be detected comparing the secretion profile of keratinocytes from patients that did not survive the thermal trauma when compared to surviving patients.

When grafts of keratinocytes were prepared after 15 days and then frozen a diminished mediator synthesis from grafts from burn patients and controls after a freeze/thaw cycle was measured. In contrast, significant higher levels of IL-1α, IL-1ß, IL-1RA were observed in cultured keratinocytes from burn injury patients after a freeze/thaw cycle and further culture for 24 hours as compared to control keratinocytes (Fig. 3A–C).

## Discussion

Tissue-engineered skin substitutes constitute a promising alternative for the permanent closure of burn wounds and in the meantime protocols have been established for the culture of large numbers of human epidermal keratinocytes [3], [14]. In particular, autologous epithelial cells grown from a single full-thickness skin biopsy have been available for nearly three decades [15]. These keratinocytes were considered solely as reconstituting elements of the skin barrier. Meanwhile it is well known that these cells secrete a multitude of biological relevant molecules which contribute to inflammatory and immunological responses and wound healing [12]. Particularly, release of IL-1 from various cells at the wound site seems to represent the initial trigger for the inflammatory reaction and serves as an autocrine, activating signal to fibroblasts and endothelial cells [16], [17].

The characterization of the secreted factors from keratinocytes could improve our understanding of their role in wound healing and – in the case of skin graft transplantation for burn injuries – improve treatments. When comparing the cellular morphology of keratinocytes in allografts made of breast reductions with autologous keratinocytes from burns removed close to the wound we observed differences in proliferative activity and differentiation as indicated by increased expression of vimentin in autologous cultured epithelial grafts and elevated KL-1 expression by keratinocytes isolated from breast reduction tissue.

Comparing the *in vitro* cytokine secretion profile of keratinocytes obtained from an area close to a full-thickness burn wound after longterm culture with normal keratinocytes significantly elevated levels of IL-2, -4, -6, -10, IFN-γ, TNF-α, TGF-β1 and GM-CSF in culture media of keratinocytes from burn patients were observed. In contrast, only minimal concentrations of IL-1β and no significant differences in IL-1α expression could be detected. While keratinocytes express the inactive pro-IL-1β, its active form is secreted [18]. Still, IL-1β was barely detectable in our experiments. IL-1α on the other hand, is barely detectable under physiologic conditions, but may be secreted by keratinocytes after tissue damage [18]. In our analyses IL-1α was detected in cultures from burn patients and controls, however no significant difference was observed after 15 days of culture.

IL-6, TNF-α, and TGF-β1 are further mediators known to exert pro-inflammatory (IL-6, TNF-a [19], [20]) and keratinocyte and fibroblast proliferating (IL-6 [21], TNF-α [22], [23], and TGF-β [24]) properties and may thus significantly contribute to the physiologic wound healing process. Keratinocytes in healthy skin do rarely express IL-6 [25]. However, cutaneous injury or physiological processes such as UVB irradiation induce increased IL-6 expression in different cell types of the dermis and in keratinocytes [26]. The significantly enhanced *in vitro* release of the potent mitogen and pro-inflammatory cytokine IL-6 from keratinocytes after severe burns may thus be important in the inflammatory event and the increased activity of the thermally affected epithelial cells with auto- and paracrine effects.

In addition, genes and proteins of IL-6 and GM-CSF show a high sequence homology and their receptor binding and signal transduction structures are uniform. This suggests common signal transduction and activation pathways [26] and may explain their comparable expression profiles in our study. Indeed, increased GM-CSF expression in human and murine skin with damaged barrier function has been demonstrated in various studies [27]–[29].

TNF-α is predominantly expressed in keratinocytes and has a profound autocrine effect on keratinocytes in wound healing. Expression of this growth factor is upregulated in keratinocytes after skin injury [30]. Surprisingly, wound healing experiments in TNF-α -deficient mice showed no abnormal wound healing phenotype [31], [32]. TNF-α acts as strong mitogen for fibroblasts [33]. However, the lack of an obvious mesenchymal phenotype in TNF-α-deficient mice suggests that this growth factor is dispensable for fibroblast proliferation and that its loss can be compensated by other EGFR ligands.

TGF-β plays an important role in wound healing as demonstrated in various in vitro and in vivo studies. The TGF-β family consists of at least five highly conserved polypeptides, with TGF-β1, -2 and -3 being the principal mammalian forms. TGF-β1 and -2 are one of the most important stimulators of collagen and proteoglycan synthesis, and affect the ECM not only by stimulating collagen synthesis but also by preventing its breakdown [34], [35]. In contrast, TGF-β3, which is predominantly induced in the later stages of wound healing, has been found to reduce connective tissue deposition [36]. Hence, overexpression of TGF-β1 and β2 has been linked to excessive scar formation [37], [38], while increased levels of TGF-β3 appear to prevent excessive scar formation. Specifically, beyond one-week, differential expression of TGF-β isoforms, receptors and activity modulators, rather than the mere presence or absence of TGF-β, may have a major role in the development of keloids and hypertrophic scarring [39]. Although, within this study only TGF-β1 was quantified, its significantly increased secretion by keratinocytes obtained from burn wounds may indeed stimulate underlying fibroblasts to produce elevated levels of ECM and by this significantly contributing to hypertrophic scar formation, commonly observed this patient population.

Noteworthy, VEGF, FGF-basic, and G-CSF were produced in relatively high quantities with no significant differences between groups. VEGF represents one of the most important pro-angiogenic factors. It accelerates wound healing by increasing neovascularization during the early period of wound healing [10], [40]. FGF-basic is produced by keratinocytes and has chemotactic effects on fibroblasts, smooth muscle cells, endothelial cells and keratinocytes [41]. Like VEGF it has pro-angiogenic properties and is essential for wound healing [42]. Clinically, local application of FGF-basic led to improved healing of diabetic ulcers [43].

However, the application of cultured keratinocytes no matter if autologous or allogenic requires the presence of a dermal component since keratinocytes do not stimulate granulation tissue formation or dermal remodelling in full-thickness wounds [44], [45]. Therefore, autologous or allogenic keratinocyte sheets are less suited for application onto inert, poorly granulating wounds (e.g., ulcers). They are more suited for the application onto well-granulating wounds in which the dermal component is already present. E.g. cultured keratinocyte sheets are applied onto large burns in a two-step procedure in which a dermal compartment (dead donor dermis) is initially applied and allowed to stabilize (develop capillaries) before applying the cultured keratinocytes [46]. Based on the delay between taking a biopsy specimen from the patient and the keratinocyte autograft becoming available, sheets of allogenic origin from unrelated donors have been used as keratinocyte allografts. Their proposed mechanisms of actions of on wound healing may indeed be mostly exerted via the secretion of the respective growth factors. However, in our study, freezing led to a diminished mediator synthesis, except for significant higher levels of IL-1α and IL-1ß, probably as a result of physical cell destruction after thawing. It thus remains questionable if cytokines and growth factors of interest survive deep-freezing.

In conclusion, epidermal keratinocytes from burn patients show an increased proliferative activity with augmented mediator release compared to keratinocytes isolated from control donors. In the future, the substitution of cellular growth-enhancing cytokines and mediators (and maybe additional cell types besides keratinocytes) in the bioengineered skin substitutes may prove promising to generate skin grafts with improved wound-healing function after burn injury.
